# N-acetyl-L-cysteine improves mitochondrial and oxidative defects in the acadian variant of fanconi syndrome

**DOI:** 10.3389/ebm.2025.10448

**Published:** 2025-05-23

**Authors:** Inas Al-Younis, Rebeca Martín-Jiménez, Mehtab Khan, Yann Baussan, Caroline Jose, Yves Thibeault, Etienne Hebert-Chatelain

**Affiliations:** ^1^ Canada Research Chair in Mitochondrial Signaling and Physiopathology, Moncton, NB, Canada; ^2^ Department of Biology, Université de Moncton, Moncton, NB, Canada; ^3^ Vitalité Health Network, Moncton, NB, Canada; ^4^ Department of Nephrology, G.-L.-Dumont University Hospital Center, Moncton, NB, Canada

**Keywords:** mitochondrial disease, antioxidant, oxidative stress, kidney disease, rare disease

## Abstract

The Acadian variant of Fanconi Syndrome (AVFS) is a rare genetic disorder characterized by renal deficiencies. AVFS is caused by a mutation to *NDUFAF6* encoding a complex I assembly factor, and leading to metabolic alterations. We confirmed that fibroblasts derived from AVFS patients have lower complex I activity, mitochondrial membrane potential and cellular respiration. These mitochondrial defects were accompanied by higher levels of 8-hydroxy-2′deoxyguanosine, malondialdehyde and carbonyl, which are markers of oxidative damage to DNA, lipids and proteins, respectively. Thus, we hypothesized that the antioxidant N-Acetyl-L-cysteine (NAC) would reduce oxidative stress and mitochondrial defects in AVFS fibroblasts. Treatment with NAC during 5 days partially restored complex I activity, mitochondrial membrane potential and cellular respiration in AVFS fibroblasts. NAC also prevented oxidative damage in AVFS fibroblasts. This work shows for the first time that the physiopathology of AVFS includes high oxidative stress. It also reveals that NAC and other antioxidant-based strategies might represent an effective pharmacological treatment for AVFS.

## Impact Statement

The Acadian variant of Fanconi Syndrome is a rare mitochondrial disease for which there is no treatment. This work identifies oxidative stress as the most important mechanism in this disorder. It also suggests that the antioxidant NAC might be useful for the treatment of patients with this syndrome. This work will be of high interest for both fundamental biologists and clinicians.

## Introduction

The Acadian variant of Fanconi Syndrome (AVFS) is a rare autosomal recessive genetic disorder characterized by defects in proximal tubular reabsorption and chronic kidney disease [1–3]. AVFS has only been reported in the Acadian population, who are descendants of the first French settlers on the East Coast of Canada. AVFS systematically progresses to end-stage renal disease and eventually requires kidney replacement therapy [2, 3]. AVFS has also been associated with other clinical features, namely pulmonary interstitial fibrosis and retinal blindness in some patients [2].

A non-coding mutation in NADH: ubiquinone oxidoreductase complex assembly factor 6 (NDUFAF6) has been reported as the cause of AVFS [1]. This mutation causes abnormal splicing of the *NDUFAF6* mRNA, which impairs the assembly of complex I (CI) of the electron transport system within mitochondria, leading to dysfunctional oxidative phosphorylation (OXPHOS) [1, 4]. OXPHOS is performed by a series of enzymatic complexes (CI to IV) which transfer high-energy electrons to O_2_ to create an electrochemical gradient across the inner mitochondrial membrane. This gradient then feeds the ATP synthase to generate ATP [5, 6]. Incomplete reduction of O_2_ during OXPHOS can lead to reactive oxygen species (ROS) such as the superoxide anion [6]. ROS can damage cellular structures and high ROS levels are associated with numerous pathological conditions, including renal diseases [7]. Thus, CI is a major entry point for electrons in OXPHOS and its dysfunction significantly increases ROS production [8]. This suggests that the mutation of NDUFAF6 could trigger high oxidative damage which would lead to the clinical symptoms of AVFS.

In this study, we hypothesized that N-Acetyl-L-cysteine (NAC), a well-known antioxidant, would alleviate AVFS symptoms. To address this, NAC was applied to fibroblasts derived from AVFS patients, in which we examined mitochondrial deficiencies and oxidative damage. The data obtained show that AVFS fibroblasts have high levels of oxidative damage which can be reduced by NAC. This work thus demonstrates for the first time the role of oxidative damage in AVFS and the potential of NAC as a pharmacological treatment in this syndrome.

## Materials and methods

All procedures were approved by the *Comité d’éthique de la recherche (CÉR) du Réseau de santé Vitalité* (#101701_2023) and the *Comité d’éthique de la recherche avec les êtres humains de l’université de Moncton* (#2223-067).

### Fibroblasts culture

Control fibroblasts were obtained from ATCC (ref. PCS-201-012). Fibroblasts from patient 1 were generated by T. Mracek (Cezch Academy of Science) and fibroblasts of patient 2 were generated at the University of Moncton. Control and patient 1 fibroblasts were used to be able to compare our data to the original work on AVFS published in 2016 [1]. The patients did not present pulmonary interstitial fibrosis at the time of the study. Information about the patients has to remain confidential considering the low number of known patients with AVFS. Patients provided written informed consent for the use of their skin biopsies.

To generate culture of fibroblasts from patients, skin biopsies were minced and then transferred in petri dish with Dulbecco’s modified Eagle’s medium (DMEM) containing 4.5 g L^−1^ glucose, 2 mM glutamine, 1 mM pyruvate, 20% (v/v) of fetal bovine serum (FBS), 100 units*mL^−1^ penicillin and 100 g*mL^−1^ streptomycin. All primary fibroblasts were maintained at 37°C in 5% CO_2_ and 95% humidity in the same culture medium.

### Chemicals

NAC (ref. A8199, Sigma-Aldrich) was first dissolved in distilled water and then in the culture medium at a concentration of 1 mM. Cells were treated for 5 days before analyses.

### Oxidative stress markers

For lipid oxidation, malondialdehyde (MDA) levels were assessed using the lipid peroxidation MDA assay kit (ref. AB233471, Abcam) with some modifications. Briefly, after cell lysis and protein normalization, thiobarbituric acid (TBA) was added to samples during 1 h at 95°C. MDA levels were detected by fluorescence using Synergy H1 microplate reader (Biotek Instrument). For protein oxidative damage, carbonyl levels were determined using the protein carbonyl content assay kit (ref. MAK094, Sigma Aldrich) following manufacturer’s instructions. DNA oxidative damage were determined by measuring the 8-OHdG levels by ELISA (ref. ab201734, Abcam) following manufacturer’s instructions.

### Complex I activity

For complex I activity, fibroblasts were harvested and protein levels were quantified. For each condition, 100 µg of protein was used in the assay as described previously [9].

### Mitochondrial membrane potential

Cells were harvested and centrifuged at 1,000*g for 5 min. Cells were then washed twice with phosphate buffered saline (pH 7.2). Cells were then incubated with 200 nM of tetramethylrhodamine methyl ester (TMRM, Ref. T668, ThermoFisher) and incubated for 30 min at 37°C in cell media. As of control, all cells were also pre-incubated with 5 µM of FCCP during 5 min at 37°C to verify that the TMRM was FCCP-sensitive. TMRM labeling was then measured using Attune cytometer (Invitrogen).

### Cellular respiration

Oxygen consumption rates (OCR) were measured using the Oxygraph-2k Oroboros system (Innsbruck, Austria), as previously described [10–12]. Briefly, cell respiration was determined at 37°C with 2 × 10^6^ intact cells in 2 mL chambers. Three different states of OCR cells were measured: (i) basal respiration, (ii) leak respiration after injection of oligomycin (2 μg mL^−1^), and (iii) uncoupled respiration after injection of FCCP (2.5 μM).

### SDS-PAGE and immunoblotting

SDS-PAGE was performed as described previously [11, 12]. Proteins were extracted in buffer containing 62.5 mM Tris–HCl, pH 6.8; 10% (v/v) glycerol, 2% (w/v) sodium dodecyl sulfate (SDS), 0.5% bromophenol blue, 2.5% (v/v) β-mercapto-ethanol) and boiled at 95°C during 5 min. Proteins were then separated at 200 V during 60 min, using 10 or 12% polyacrylamide gel containing 0.35% (V/V) of 2,2,2-trichloroethanol for total protein staining, as previously described [13].

After electrophoresis, proteins were transferred to polyvinylidene difluoride (PVDF) membranes. Membranes were blocked for 1 h in 50 mM Tris–Cl, pH 7.6; 150 mM NaCl, 0.1% Tween, containing 5% BSA or 5% skimmed milk. PVDF membranes were incubated with primary antibodies overnight at 4°C. Protein immunodetection was performed using primary antibodies directed against NDUFA9 (ab14713, Abcam), NDUFAF6 (sc-17091, Santa Cruz), SDHA (ab14715, Abcam), UQCRC2 (ab14742, Abcam), TOM20 (sc-17764, Santa Cruz). Then, PVDF membranes were incubated for 1 h with appropriate peroxidase-conjugated antibodies. Finally, immunoblots were visualized by chemiluminescence using the ChemiDoc Touch imaging system (Biorad, USA).

### Statistical analyses

Data are presented as mean ± SEM and were statistically analyzed using one-way or two-way ANOVA followed by Tukey *post hoc* test with GraphPad Prism 9. In figures, the datapoints correspond to experiments conducted independently on different days. The results of *post hoc* tests are shown with letters: datapoints with different letters are statistically different (p < 0.05) whereas datapoints with the same letters are not statistically different (p > 0.05). For instance, a datapoint labelled with the letter a is statistically different from datapoints marked with the letters b or bc, whereas it is not statistically different from datapoints labelled with the letters a or ab.

## Results

**FIGURE 1:**
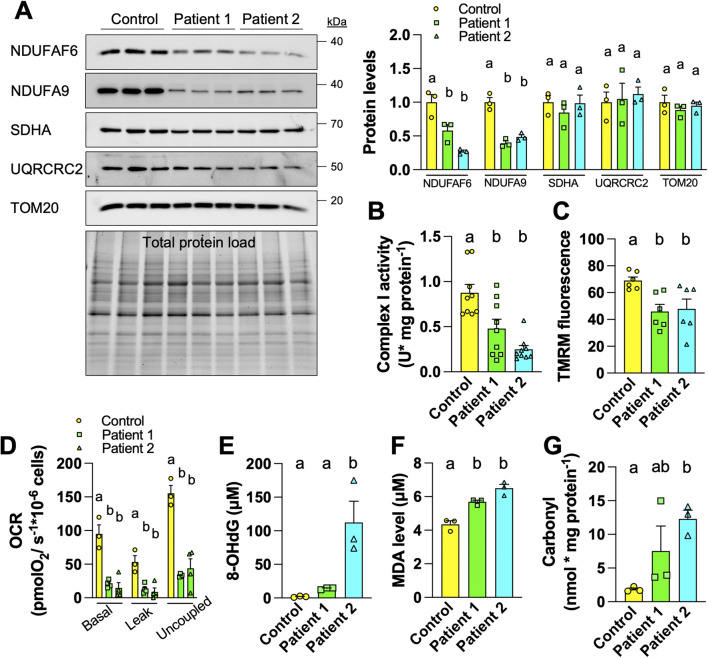
Fibroblasts derived from Acadian variant of Fanconi syndrome (AVFS) patients have mitochondrial deficits and oxidative damage. **(A)** Immunoblotting and quantification of mitochondrial proteins in fibroblasts derived from control or AVFS patients showing lower levels of NDUFAF6 and the complex I subunit NDUFA9 but not of SDHA, UQRCRC2 and TOM20. **(B)** The enzymatic activity of complex I is decreased in AVFS fibroblasts (n = 9). **(C)** Fluorescence of the mitochondrial membrane potential-dependent TMRM is decreased in AVFS fibroblasts (n = 6). **(D)** Oxygen consumption rates (OCR) at different respiratory states indicating lower mitochondrial metabolism in AVFS fibroblasts (n = 3-4). **(E–G)** Levels of **(E)** 8-hydroxy-2′-deoxyguanosine (8-OHdG), **(F)** malondialdehyde (MDA) and **(G)** carbonyl, which indicate oxidative damage on DNA, lipid and protein, respectively, are increased in AVFS fibroblasts (n = 3). Data are presented as mean +- SEM. Data with different letters are statistically different, as measured by one way ANOVA followed by post-hoc Tukey test.

We first compared the mitochondrial physiology in fibroblasts from control and AVFS patients. The control and patient 1 fibroblasts are the same cells analyzed in the original work on AVFS [1], whereas the fibroblasts of patient 2 were generated for this study. We characterized the molecular defects associated with the syndrome. AVFS is caused by a mutation in the CI assembly factor NDUFAF6, reducing the levels of the NDUFAF6 protein, thereby impairing complex I assembly and activity [1]. To confirm this, we compared levels of different mitochondrial proteins between fibroblasts derived from one control and two AVFS patients by immunoblotting. The data obtained show reduced levels of NDUFAF6 and of the complex I subunit NDUFA9 (Figure 1A). However, levels of CII subunit SDHA, CIII subunit UQRCRC2, and TOM20 were similar between control and AVFS fibroblasts (Figure 1A), confirming that AVFS specifically involves CI deficiencies and is not linked to a global reduction of mitochondrial mass, as previously reported [1].

In parallel to decreased levels of CI-related proteins (Figure 1A), we also observed reduced enzymatic activity of CI in AVFS fibroblasts (Figure 1B). To evaluate the impact on OXPHOS, we then measured mitochondrial membrane potential and oxygen consumption. Labeling with the mitochondrial membrane potential-dependent dye TMRM showed that AVFS fibroblasts have altered OXPHOS as compared to the control fibroblasts (Figure 1C). Similarly, OCR of 3 different respiratory states were also decreased in AVFS fibroblasts (Figure 1D). These data confirm the presence of mitochondrial dysfunction in AVFS fibroblasts, as observed previously [1].

Alterations in CI assembly and activity are a major cause of oxidative stress [8]. To address whether the AVFS-related CI deficiencies result in higher oxidative damage, we examined levels of 8-hydroxy-2′deoxyguanosine (8-OHdG), malondialdehyde (MDA), and carbonyl, which are well-known biomarkers for oxidative damage to DNA, lipids and proteins, respectively. Strikingly, levels of 8-OHdG, MDA and carbonyl were all increased in AVFS fibroblasts (Figures 1E–G). These findings indicate that AVFS involves oxidative stress.

**FIGURE 2:**
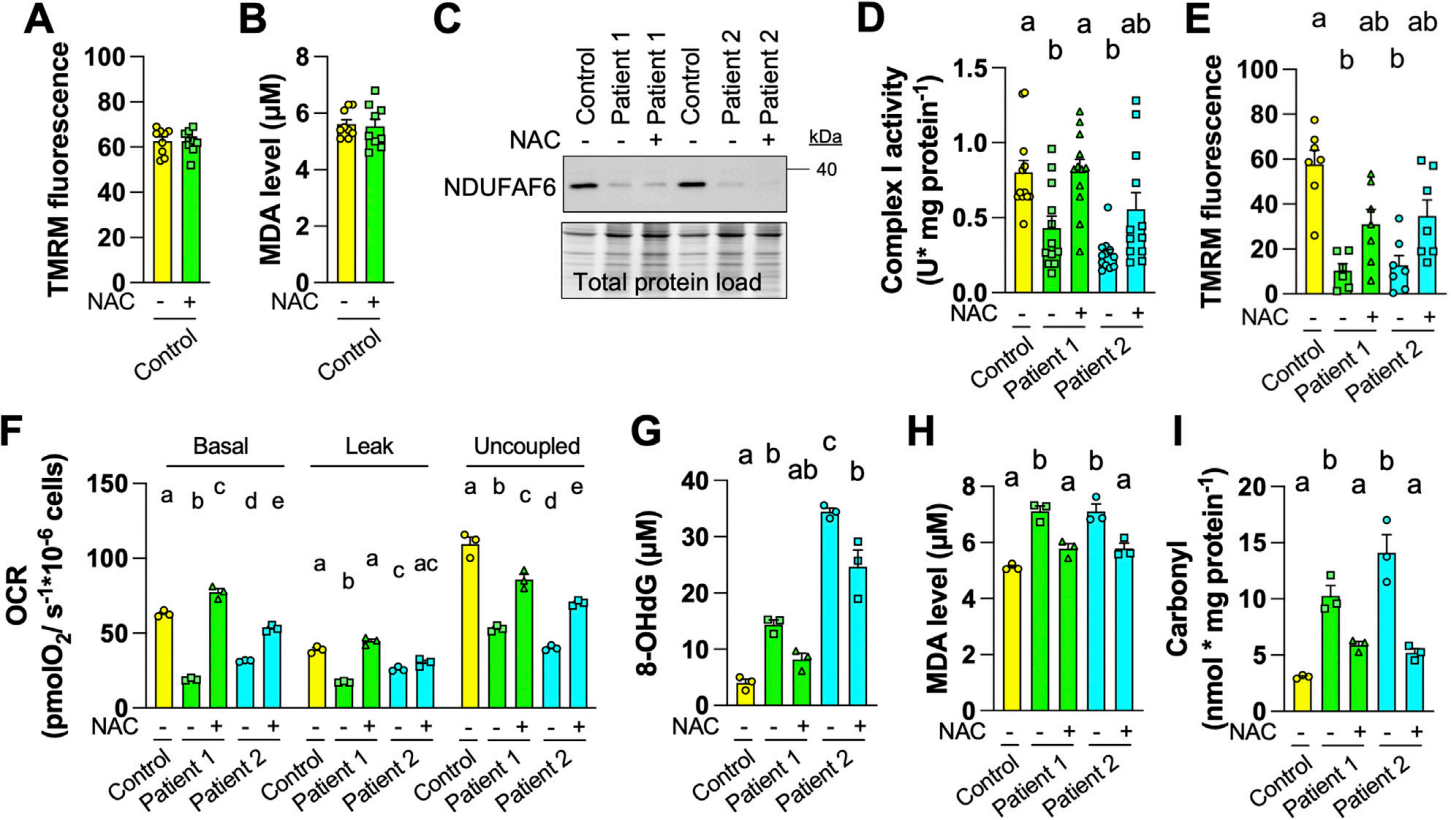
Treatment with the antioxidant N-Acetyl-L-cysteine (NAC) reverses oxidative damage in fibroblasts derived from Acadian variant of Fanconi syndrome (AVFS) patients. **(A)** TMRM fluorescence in control fibroblasts do not change after treatment with NAC (1 mM, 5 days, n = 9). **(B)** MDA levels in control fibroblasts do not change after treatment with NAC (n = 9). **(C)** Representative immunoblotting (n = 3) of NDUFAF6 in control and AVFS fibroblasts with vehicle or NAC, showing that NAC does not rescue levels of NDUFAF6. **(D)** The enzymatic activity of complex I is partly rescued in AVFS fibroblasts upon treatment with NAC (n = 12). **(E)** TMRM fluorescence is partly rescued in AVFS fibroblasts treated with NAC (n = 6-7). **(F)** Oxygen consumption rates (OCR) at different respiratory states are partly rescued upon treatment with NAC (n = 3) **(G–I)** Levels of **(G)** 8-hydroxy-2′-deoxyguanosine (8-OHdG), **(H)** malondialdehyde (MDA) and **(I)** carbonyl, which indicate that oxidative damage on DNA, lipid and protein, respectively, are partly rescued in AVFS fibroblasts treated with NAC (n = 3). Data are presented as mean +- SEM. Data with different letters are statistically different, as measured by one way ANOVA followed by post-hoc Tukey test.

We hypothesized that a treatment with an antioxidant, such as NAC, would alleviate the mitochondrial and oxidative alterations observed in AVFS fibroblasts. To address this, fibroblasts were treated with NAC (1 mM) for 5 days. This treatment had no effect on TMRM fluorescence and MDA levels in control fibroblasts (Figures 2A,B). Treatment of AVFS fibroblasts with NAC did not rescue NDUFAF6 levels (Figure 2C), but almost completely restored CI activity (Figure 2D) and mitochondrial membrane potential (Figure 2E). The NAC treatment fully rescued basal and leak OCR, but partially rescued uncoupled respiration in AVFS fibroblasts (Figure 2F). Levels of 8-OHdG were partially restored, whereas levels of MDA and carbonyl were completely restored in AVFS fibroblasts treated with NAC (Figures 2G–I). These findings indicate that the treatment with NAC is sufficient to reverse most of the oxidative damage induced by AVFS, and restore, at least partly, the mitochondrial deficiencies in fibroblasts derived from patients.

## Discussion

The aim of this work was to examine whether fibroblasts from AVFS patients have higher oxidative stress and test the potential beneficial effect of the antioxidant NAC to rescue the cellular and molecular defects linked to AVFS. Our findings demonstrate that AVFS fibroblasts from two different patients have higher levels of oxidative damage than control fibroblasts. Our findings also indicate that treatment with NAC can rescue the mitochondrial dysfunction and the oxidative damage associated to AVFS. This work suggests that NAC might be considered as promising treatment for AVFS.

NAC is a derivative of the amino acid cysteine and its antioxidant effect originates from its ability to promote the concentration of glutathione, an important thiol compound regulating redox potential within cells [14]. NAC is considered as a safe and inexpensive dietary supplement with limited side-effects, and is available over-the-counter in several countries [14]. It has received FDA approval for treatment of hepatotoxic dose of acetominophen [14]. NAC was also approved more recently for conditions with abnormal mucus secretion, including pneumonia and bronchitis [14]. NAC is used as short- and long-term treatment in several diseases related to both acute and chronic kidney impairments. For instance, short-term NAC treatment has been used in contrast-induced acute kidney injury, a condition in which kidney function is acutely altered after intravascular administration of iodinated contrast media for imaging purposes [15]. However, no global positive effects of NAC have been observed in this condition [16]. This could be due to the low bioavailability of the antioxidant upon oral administration [17–20]. However, various types of treatment with NAC appears to improve eGFR and reduce cardiovascular events in chronic kidney disease (CDK) [15]. For instance, patients with CDK treated with a chronic NAC regimen (600 mg of NAC orally twice daily during 3 years) had improved eGFR as compared to non-NAC users [19]. Overall, this suggest that chronic regimen with NAC could improve the renal function of AVFS patients.

Our findings suggest that oxidative stress is an important component within the physiopathology of AVFS since the most part of cellular defects in AVFS were rescued by NAC. For instance, the CI activity was almost completely rescued by NAC, indicating that the CI enzymatic alterations caused by the AVFS-related mutation of NDUFAF6 are likely a consequence of oxidative damage on CI itself. Although this work is the first to report increased oxidative damage in AVFS, oxidative stress is commonly observed in mitochondrial diseases and other pathologies involving secondary mitochondrial dysfunctions, including neurodegenerative diseases [21]. In turn, NAC has been used to reverse oxidative stress in several pathological conditions related to mitochondrial dysfunctions [22–24]. For instance, it has been shown that NAC decreases levels of ROS and improve ATP production as well as mitochondrial membrane potential in fibroblasts derived from patients with different mitochondrial diseases [24]. NAC also delays the progression of motor deficits and improves mitochondrial functions in the R6/1 mouse models of Huntington’s disease [25]. Overall, NAC improves mitochondrial function in cellular and animal models of various disorders involving mitochondrial dysfunction. However, this work is the first to demonstrate that NAC can be beneficial in AVFS patients.

It will be important to examine whether the positive effects of NAC observed in AVFS fibroblasts can be translated in patients. Indeed, fibroblasts from patients might not perfectly mimic the renal and lung cell types affected in AVFS since mitochondrial physiology varies across tissue and cell types [26–29]. Our findings could also be biased by the comparison with control fibroblasts obtained from ATCC, which potentially introduced variability due to differences in donor age, sex, and genetic background. Thus, the physiopathology of AVFS and the effect of NAC or other antioxidants should also be examined in patient-derived renal cells.

In conclusion, NAC represents a promising option for the treatment of AVFS since it rescued oxidative damage and mitochondrial dysfunction of AVFS fibroblasts. More studies should explore the potential beneficial effect of NAC and other antioxidant-based strategies in AVFS patients.

## Data Availability

The raw data supporting the conclusions of this article will be made available by the authors, without undue reservation.
